# Safety and immunogenicity of co-administration of meningococcal type A and measles–rubella vaccines with typhoid conjugate vaccine in children aged 15–23 months in Burkina Faso

**DOI:** 10.1016/j.ijid.2020.10.103

**Published:** 2021-01

**Authors:** Sodiomon B. Sirima, Alphonse Ouedraogo, Nouhoun Barry, Mohamadou Siribie, Alfred B. Tiono, Issa Nébié, Amadou T. Konaté, Gloria Damoaliga Berges, Amidou Diarra, Moussa Ouedraogo, Issiaka Soulama, Alimatou Hema, Shrimati Datta, Yuanyuan Liang, Elizabeth T. Rotrosen, J. Kathleen Tracy, Leslie P. Jamka, Kathleen M. Neuzil, Matthew B. Laurens

**Affiliations:** a Groupe de Recherche Action en Santé, Ouagadougou, Burkina Faso, West Africa; b Center for Vaccine Development and Global Health, University of Maryland School of Medicine, Baltimore, MD, USA

**Keywords:** Typhoid conjugate vaccine, Meningococcal vaccines, Measles–rubella vaccine, Co-administration, Sub-Saharan Africa, Burkina Faso

## Abstract

**Objectives:**

The World Health Organization pre-qualified single-dose typhoid conjugate vaccine (TCV) and requested data on co-administration with routine vaccines. The co-administration of Typbar TCV (Bharat Biotech International) with routine group A meningococcal conjugate vaccine (MCV-A) and measles–rubella (MR) vaccine was tested.

**Methods:**

This was a double-blind, randomized controlled trial performed in Ouagadougou, Burkina Faso. Children were recruited at the 15-month vaccination visit and were assigned randomly (1:1:1) to three groups. Group 1 children received TCV plus control vaccine (inactivated polio vaccine) and MCV-A 28 days later; group 2 children received TCV and MCV-A; group 3 children received MCV-A and control vaccine. Routine MR vaccine was administered to all participants. Safety was assessed at 0, 3, and 7 days after immunization, and unsolicited adverse events and serious adverse events were assessed for 28 days and 6 months after immunization, respectively.

**Results:**

A total of 150 children were recruited and vaccinated. Solicited symptoms were infrequent and similar for TCV and control recipients, as were adverse events (group 1, 61.2%; group 2, 64.0%; group 3, 68.6%) and serious adverse events (group 1, 2.0%; group 2, 8.0%; group 3, 5.9%). TCV generated robust immunity without interference with MCV-A vaccine.

**Conclusions:**

TCV can be safely co-administered at 15 months with MCV-A without interference. This novel study on the co-administration of TCV with MCV-A provides data to support large-scale uptake in sub-Saharan Africa.

## Highlights

• This is the first study on the co-administration of typhoid conjugate vaccine (TCV) in West Africa.
• Co-administration of TCV with routine vaccines in a typhoid-endemic country was successful.
• TCV was safely co-administered at 15 months with group A meningococcal conjugate vaccine.
• Single-dose TCV was immunogenic in 15-month-old children.
• There was no safety signal related to TCV vaccination or co-administration.

## Introduction

Typhoid fever is an acute infection caused by exposure to *Salmonella enterica* serovar Typhi (*Salmonella* Typhi) in food or water contaminated with human faeces. In endemic areas, typhoid fever incidence is highest in school-age children, but it is increasingly documented in children under five (Kim et al., 2019). Each year, nearly 11 million cases and more than 116,000 related deaths occur from typhoid fever worldwide, mostly among children and young adults in Asia and sub-Saharan Africa (GBD 2017 Typhoid and Paratyphoid Collaborators, 2019). Estimating typhoid fever incidence is challenging in low- and middle-income countries due to the non-specific clinical presentation and limited access to blood culture, the most reliable diagnostic test.

The Typhoid Fever Surveillance in Africa Program (TSAP) measured invasive *Salmonella* bloodstream infection incidence across 13 sites in 10 African countries from September 2011 to December 2013 (Al-Emran et al., 2016). The TSAP documented an overall adjusted incidence of 10–100 cases per 100,000 people, two to three times higher than previous estimates (Crump et al., 2004). Incidence was highest in children 2–14 years old. Importantly, 47% of *Salmonella* Typhi isolates were multidrug-resistant (Al-Emran et al., 2016). Outbreaks of antibiotic-resistant typhoid fever have recently occurred throughout sub-Saharan Africa, including Malawi, Uganda, Zimbabwe, Zambia, and the Democratic Republic of the Congo (Blum et al., 2014, Kabwama et al., 2017, Polonsky et al., 2014, Hendriksen et al., 2015, Muyembe-Tamfum et al., 2009).

Before 2017, two typhoid vaccines were available (World Health Organization, 2008), an oral, live, attenuated Ty21a vaccine licensed for children 6 years and older and an injectable Vi capsular polysaccharide vaccine licensed for children 2 years and older. In 2013, a new typhoid vaccine conjugated to a tetanus toxoid carrier protein (Typbar TCV) was licensed in India for children as young as 6 months of age. In December 2017, the World Health Organization (WHO) prequalified the vaccine, facilitating introduction in low-income countries. The WHO recommends typhoid conjugate vaccine (TCV) for children 6 months and older in typhoid-endemic countries, acknowledging that efficacy trials and co-administration studies should be prioritized (World Health Organization, 2017, World Health Organization, 2018). Interim results from a randomized controlled trial in Nepal demonstrated over 81% efficacy in children 9 months to 16 years of age (Shakya et al., 2019), and trials are underway in Malawi and Bangladesh (Meiring et al., 2019, Theiss-Nyland et al., 2019). While co-administration of TCV with measles–rubella (MR) vaccine has been studied (Bhutta et al., 2014), TCV has not been tested with routine immunizations in West Africa, where typhoid is endemic and where group A meningococcal conjugate vaccine (MCV-A), which also contains a tetanus toxoid carrier protein, is routinely given at 15 months of age as part of the Expanded Programme on Immunization (EPI). These data are essential for large-scale uptake of TCV in sub-Saharan Africa.

This study is part of the work of the Typhoid Vaccine Acceleration Consortium (TyVAC), which aims to generate evidence to support TCV introduction as part of an integrated approach to reduce the burden of typhoid in endemic countries. TCV co-administration with EPI vaccines was assessed among children 9 through 11 months of age and 15 through 23 months of age in Burkina Faso. Results from co-administration with yellow fever and MR vaccines at the 9-month EPI visit will be reported separately. This study was performed to assess the safety and immunogenicity of TCV when co-administered with routine MR vaccine and with and without MCV-A, in children at their routine 15-month EPI visit in Burkina Faso (Neuzil et al., 2019). The immunogenicity of MCV-A vaccine when administered with and without TCV was also assessed.

## Methods

### Study design

This was a double-blind, randomized controlled trial conducted in the paediatric outpatient clinic of Schiphra Protestant Hospital, an urban hospital in Ouagadougou, Burkina Faso. The study protocol has been published previously (Laurens et al., 2019).

### Participants

Healthy children were recruited at their routine 15-month vaccination visit. Groups of parents and caregivers were given general information about the study upon arrival. Information was provided and preliminary questions were answered orally in French and local languages. The study clinicians met with each interested parent/caregiver, answered questions, and obtained written informed consent. All forms were in French or available via oral translation into local languages with independent witness certification. Each parent/guardian either signed or provided a thumbprint on the consent form.

Participants were eligible for enrolment if they were healthy and 15–23 months of age inclusive, and if their parent/guardian resided in the study area. Participants were excluded if they had any of the following: a known hypersensitivity to any vaccine component; previously received a typhoid vaccine; history of severe allergic reaction or chronic illness; severe malnutrition; recent or planned receipt of any investigational product during the study period; receipt of blood products in the previous 6 months; known human immunodeficiency virus infection or exposure, or immunosuppressive condition; receipt of systemic immunosuppressive medication, including corticosteroids; or any other condition determined by the investigators to interfere with the evaluation of the vaccine or to present a health risk related to study participation. Participants were temporarily excluded for 48 h if they presented with fever or a history of fever within the previous 24 h. Participants received either TCV or the control vaccine (inactivated polio vaccine, IPV). A full list of inclusion and exclusion criteria is available in the study protocol (Laurens et al., 2019).

### Randomization and masking

Using a computer-generated algorithm, the participants were assigned randomly to three groups in a 1:1:1 ratio with stratified block randomization and varying block sizes from 6 to 12. The participants were assigned to one of the following groups: group 1 patients received TCV plus control vaccine (IPV) and MCV-A 28 days later; group 2 patients received TCV and MCV-A; group 3 patients received MCV-A and control vaccine. After the study clinicians had completed screening and eligibility procedures, the participants were assigned a unique treatment allocation code generated by the study biostatistician and programmed into the Research Electronic Data Capture (REDCap) study database. The randomization module was only accessible to the unblinded study pharmacist responsible for vaccine preparation. TCV and IPV are identical in colour and volume (0.5 ml) and vaccine syringes were prepared behind a privacy screen to maintain blinding of participants and parents/caregivers to treatment assignment. An unblinded study nurse, who had no other role in the clinical trial and no contact with participants after vaccination, administered the vaccine. Study staff who assessed the outcomes were blinded to the group assignment. Study investigators analysing the data were unblinded to group assignment.

### Procedures

The TCV vaccine (Typbar TCV; Bharat Biotech International, Hyderabad, India) used in this study consists of 25 μg of Vi polysaccharide conjugated to a non-toxic tetanus toxoid protein carrier. The IPV control vaccine (IMOVAX POLIO; Sanofi Pasteur, Lyon, France) consists of 40 d-antigen units (DU) of poliovirus type 1, 8 DU of poliovirus type 2, and 32 DU of poliovirus type 3 preserved in 2-phenoxyethanol (5 mg/mL). TCV 0.5 ml or IPV 0.5 ml was injected intramuscularly into the anterolateral thigh or upper deltoid of each participant. Vaccinations were administered in the paediatric outpatient clinic of Schiphra Protestant Hospital in Ouagadougou, Burkina Faso. Trained local study staff who were not involved in participant follow-up or adverse event (AE) assessment, administered the injections. On study day 0, group 1 participants received TCV in the left deltoid, IPV in the left thigh, and MR in the right deltoid; group 2 participants received TCV in the left thigh, MR in the right deltoid, and MCV-A in the left deltoid; and group 3 participants received IPV in the left thigh, MR in the right deltoid, and MCV-A in the left deltoid. On study day 28, group 1 participants received MCV-A in the left deltoid. Potential baseline *Plasmodium falciparum* malaria co-infection was assessed by microscopic evaluation of a thick blood smear collected on day 0.

The participants were monitored on site for serious reactions for 30 min post-vaccination. Home or clinic visits occurred on days 3 and 7 post-vaccination to solicit local and systemic AEs. Study personnel were always available for unscheduled visits (Laurens et al., 2019). Unsolicited AEs were recorded until day 28 and serious AEs (SAEs), as defined in the study protocol (Laurens et al., 2019), were recorded until day 180. Blood was drawn for protocol-specified laboratory assessments on day 0 (malaria and immunogenicity) and 28 (immunogenicity). Grading of AEs was based on the United States Food and Drug Administration (FDA) guidelines for vaccine clinical trials (US Food and Drug Administration, 2007).

Anti-Vi antibody responses were measured using the VaccZyme *Salmonella* Typhi Vi immunoglobulin G (IgG) ELISA kit (The Binding Site Group Ltd, Birmingham, UK). Anti-tetanus antibody responses were measured using the anti-tetanus toxoid ELISA (IgG) kit (Euroimmun AG, Lübek, Germany). Both Vi IgG and anti-tetanus antibody testing were done at the Groupe de Recherche Action en Santé (GRAS) laboratories in Ouagadougou, Burkina Faso. Anti-meningitis A antibodies were measured by serum bactericidal antibody (SBA) concentration at the Meningococcal Reference Unit, National Infection Service, Public Health England, Manchester, UK (Lucidarme et al., 2019). Blood smears were examined according to standard procedures by two independent technicians with documented training and expertise in malaria microscopy.

### Outcomes

Primary study outcomes included the proportion of participants who experienced the following: (1) AEs in the first 30 min after vaccination and for 7 days after vaccination, (2) other non-serious AEs up to 28 days after vaccination, and (3) SAEs within 6 months of vaccination. Secondary outcomes included anti-Vi IgG antibody titre and SBA titre against *Neisseria meningitidis* serogroup A at day 28. Participants who attained a ≥4-fold rise in anti-Vi IgG antibody from day 0 to day 28 and an SBA titre of ≥128 (reciprocal of the final serum dilution) were considered seroprotected against typhoid and *N. meningitidis*, respectively. Exploratory study outcomes included tetanus toxoid antibody levels at day 0 and 28 and *Plasmodium falciparum* parasitaemia associated with a decreased immunogenic response to vaccination. Participants with anti-tetanus IgG levels of 0.1 to <1.0 IU/mL were considered to have short-term immunity, while those with levels ≥1.0 were considered to have long-term immunity.

### Statistical analysis

The sample size of 50 per group, with up to 10% loss to follow-up, was calculated to achieve 80% power to detect a minimum increase of 0.23 in AE incidence assuming 10% incidence in controls and using a two-sided *z*-test with unpooled variance and a significance level of 5%. In addition, a sample size of 45 produces a two-sided 95% confidence interval with a width equal to 0.196 (i.e., 3.1%–22.7%) when the sample proportion is 10% using the Clopper–Pearson exact method. All participants who received TCV and/or IPV were included in safety analyses, and participants who received all assigned vaccinations were included in the analysis detailed in the protocol (Laurens et al., 2019). Participants in group 1 did not receive MCV-A vaccination on day 0; therefore, their SBA titres were not assessed. The sample size was calculated using PASS 15 and the study results were analysed using SAS software version 9.4 (Copyright 2016; SAS Institute Inc.). As all vaccines in this study are approved and prequalified by the WHO, no data monitoring committee oversaw the study. The trial is registered at ClinicalTrials.gov, Identifier NCT03614533.

### Role of the funding source

The project funder had no role in the study design, data collection, data analysis, data interpretation, or writing of the article. The corresponding author had full access to all of the data in the study and final responsibility for the decision to submit for publication.

## Results

**Figure 1:**
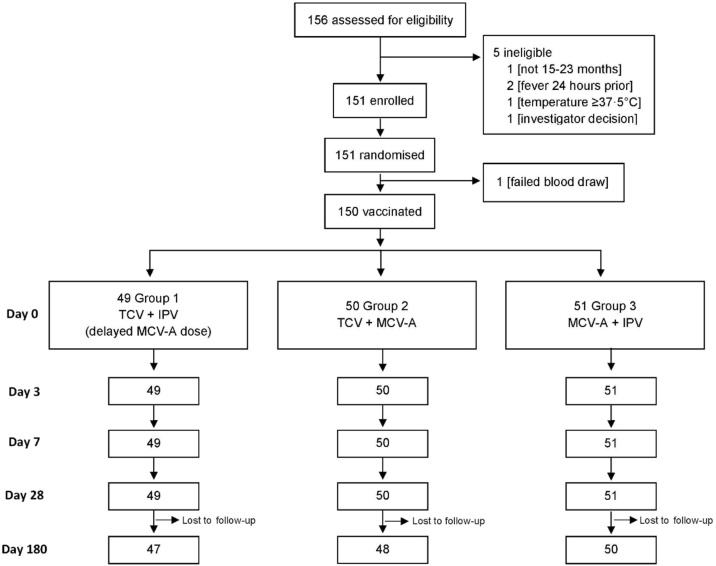
Disposition of participants (CONSORT flow diagram).

From December 3, 2018 to February 18, 2019, a total of 156 children were assessed for eligibility, 151 of whom were enrolled (Figure 1). Overall, 49 participants were enrolled in group 1, 51 in group 2, and 51 in group 3. One enrolled participant in group 2 was not vaccinated because of inability to collect a baseline blood sample.

Table 1 summarizes the baseline characteristics of study participants at enrolment. Most participants were approximately 16 months of age. A baseline meningococcal A SBA titre was detectable in a greater number of participants in group 2 and baseline Vi titre was detectable in five to seven children in each group. Three participants were positive for *P. falciparum* malaria on study day 0.

**Table 1:** Demographics and baseline characteristics of study participants at enrolment.

	Group 1 TCV + IPV (delayed MCV-A)	Group 2 TCV + MCV-A	Group 3 MCV-A + IPV
Enrolled	49	51	51
Vaccinated	49	50	51

Sex			
Female	19 (38.8%)	23 (46.0%)	31 (60.8%)
Male	30 (61.2%)	27 (54.0%)	20 (39.2%)
Age (months)	16.4 ± 1.7	16.1 ± 1.7	15.7 ± 1.2
Baseline meningococcal A SBA titre (≥4)	NA	24 (48.0%)	10 (19.6%)
Baseline Vi titre (≥7.4 EU/mL)	7 (14.3%)	5 (10.0%)	6 (11.8%)
Baseline tetanus titre (≥0.001 IU/mL)	47 (96.0%)	50 (100%)	51 (100%)
Baseline malaria positive	2 (4.1%)	1 (2.0%)	0 (0%)

Data are *n* (%) or mean ± standard deviation. *n* = number of participants. EU, ELISA units; IPV, inactivated polio vaccine; IU, international units; MCV-A, group A meningococcal conjugate vaccine; NA, not applicable; SBA, serum bactericidal antibody; TCV, typhoid conjugate vaccine.

The vaccinations were well-tolerated. There were no reactions within the first 30 min. Most participants reported no local or systemic AEs after vaccination (Table 2). Local reactions were reported in only two participants, both in group 3 (data not shown) and both were mild (pain/tenderness and swelling). Systemic reactions were all mild. Unsolicited AEs were likewise uncommon and none deemed related to vaccination. SAEs were reported for one participant in group 1, four in group 2, and three in group 3, and most were gastroenteritis. None were deemed related to vaccination. No deaths occurred.

**Table 2:** Summary of safety parameters by group, paricipant level.

		Group 1 TCV + IPV (delayed MCV-A)	Group 2 TCV + MCV-A	Group 3 MCV-A + IPV
		(*n* = 49)	(*n* = 50)	(*n* = 51)
Systemic reactions				
Day 0	Fever	3 (6.1, 1.3–16.9)	1 (2.0, 0.1–10.7)	3 (5.9, 1.2–16.2)
	Irritability	0 (0.0, 0.0–7.3)	0 (0.0, 0.0–7.1)	0 (0.0, 0.0–7.0)
	Any systemic reaction	3 (6.1, 1.3–16.9)	1 (2.0, 0.1–10.7)	3 (5.9, 1.2–16.2)
Day 3	Fever	1 (2.0, 0.1–10.9)	1 (2.0, 0.1–10.7)	2 (3.9, 0.5–13.5)
	Irritability	1 (2.0, 0.1–10.9)	0 (0.0, 0.0–7.1)	1 (2.0, 0.1–10.5)
	Any systemic reaction	2 (4.1, 0.5–14.0)	1 (2.0, 0.1–10.7)	3 (5.9, 1.2–16.2)
Day 7	Fever	0 (0.0, 0.0–7.3)	2 (4.0, 0.5–13.7)	2 (3.9, 0.5–13.5)
	Irritability	0 (0.0, 0.0–7.3)	1 (2.0, 0.1–10.9)	0 (0.0, 0.0–7.0)
	Any systemic reaction	0 (0.0, 0.0–7.3)	2 (4.0, 0.5–13.7)	2 (3.9, 0.5–13.5)
Days 0, 3, and 7	Any systemic reaction	5 (10.2, 3.4–22.2)	3 (6.0, 1.3–16.6)	8 (15.7, 7.0–28.6)

Adverse events, unsolicited				
	Conjunctivitis	1 (2.0, 0.1–10.9)	2 (4.0, 0.5–13.7)	4 (7.8, 2.2–18.9)
	Cough	3 (6.1, 1.3–16.9)	5 (10.0, 3.3–21.8)	7 (13.7, 5.7–26.3)
	Diarrhoea	13 (26.5, 15.0–41.1)	12 (24.0, 13.1–38.2)	12 (23.5, 12.8–37.5)
	Fever with no source	4 (8.2, 2.3–19.6)	13 (26.0, 14.6–40.3)	7 (13.7, 5.7–26.3)
	Other rash or skin disorder	2 (4.1, 0.5–14.0)	2 (4.0, 0.5–13.7)	5 (9.8, 3.3–21.4)
	Upper respiratory illness	14 (28.6, 16.6–43.3)	17 (34.0, 21.2–48.8)	19 (37.3, 24.1–51.9)
	Vomiting	4 (8.2, 2.3–19.6)	8 (16.0, 7.2–29.1)	5 (9.8, 3.3–21.4)
	Other	3 (6.1, 1.3–16.9)	6 (12.0, 4.5–24.3)	2 (3.9, 0.5–13.5)
	Any adverse event	30 (61.2, 46.2–74.8)	32 (64.0, 49.2–77.1)	35 (68.6, 54.1–80.9)

Serious adverse events				
	Diarrhoea/gastroenteritis	1 (2.0, 0.1–10.9)	3 (6.0, 1.3–16.6)	1 (2.0, 0.1–10.5)
	Hyperthermia	0 (0.0, 0.0–7.3)	0 (0.0, 0.0–7.1)	1 (2.0, 0.1–10.5)
	Malaria	0 (0.0, 0.0–7.3)	0 (0.0, 0.0–7.1)	1 (2.0, 0.1–10.5)
	Pneumonia	0 (0.0, 0.0–7.3)	1 (2.0, 0.1–10.7)	0 (0.0, 0.0–7.0)
	Any serious adverse event	1 (2.0, 0.1–10.9)	4 (8.0, 2.2–19.2)	3 (5.9, 1.2–16.2)

Data are *n* (%, 95% confidence interval). *n* = number of participants. IPV, inactivated polio vaccine; MCV-A, group A meningococcal conjugate vaccine; TCV, typhoid conjugate vaccine.

**Figure 2:**
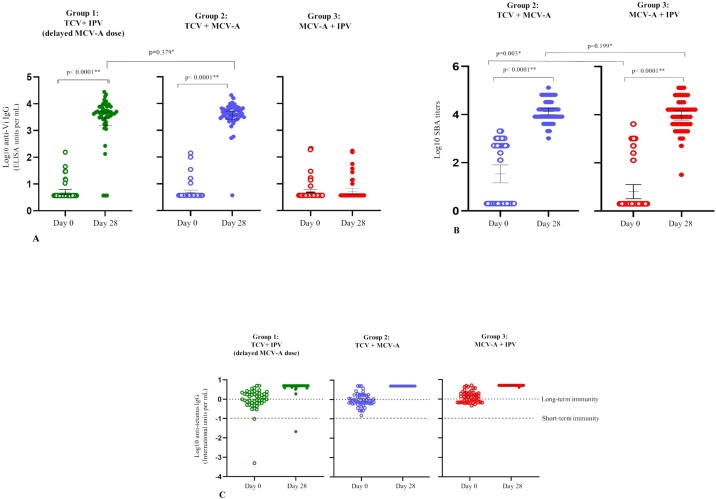
(A) Anti-Vi IgG antibody titres before vaccination (day 0) and 28 days after vaccination. (B) Serum bactericidal antibody titres before vaccination (day 0) and 28 days after vaccination. (C) Anti-tetanus IgG titres before vaccination and 28 days after vaccination. *Using the two-sample *t*-test with unequal variances on log_10_ transformed data. **Using the paired *t*-test on log_10_ transformed data.

Anti-Vi antibody titres for groups 1, 2, and 3 were low on day 0 and significantly higher post-vaccination for groups 1 and 2, who received TCV (Table 3 and Figure 2A). The anti-Vi antibody geometric mean titre (GMT) before vaccination was 5.0, 4.7, and 4.8 ELISA units (EU)/mL for groups 1, 2, and 3, respectively. Post-vaccination, these levels rose to 2754.1 EU/mL for group 1 and 3707.3 EU/mL for group 2, while remaining relatively unchanged at 5.3 EU/mL for group 3, the group of participants who did not receive TCV. Except for three participants in group 1 and two participants in group 2, all participants vaccinated with TCV achieved seroconversion. Analyses by sex showed similar results (Supplementary Material Table A1).

**Table 3:** Anti-Vi IgG antibody immunogenicity before vaccination (day 0) and 28 days after vaccination.

	Group 1 TCV + IPV (delayed MCV-A)	Group 2 TCV + MCV-A	Group 3 MCV-A + IPV
	(*n* = 48)^a^	(*n* = 50)	(*n* = 51)
Seroconversion (≥4-fold rise from day 0 to 28 days after vaccination)	44^b^ (93.6%, 82.5–98.7%)	48 (96.0%, 86.3–99.5%)	2 (3.9, 0.5–13.5%)
Day 0 geometric mean titre	5.0 (4.0–6.3)	4.7 (3.7–5.8)	4.8 (3.8–6.2)
Day 28 geometric mean titre	2754.1^b^ (1537.3–4934.1)	3707.3 (2632.0–5222.0)	5.3 (4.1–6.9)

Data are *n* (%, 95% confidence interval) or mean (95% confidence interval). *n* = number of participants. IPV, inactivated polio vaccine; MCV-A, group A meningococcal conjugate vaccine; TCV, typhoid conjugate vaccine.

a One participant excluded in per-protocol immunogenicity analysis for late day 28 visit.

b One participant missing titre, *n* = 47.

SBA titres for groups 2 and 3 were low on day 0 and significantly higher post-vaccination (Table 4 and Figure 2B). Except for one participant in group 3, all participants in groups 2 and 3 had seroprotective SBA titres post-vaccination. Analyses by sex showed similar results (Supplementary Material Table A2).

**Table 4:** Anti-meningococcal A antibody immunogenicity before vaccination (day 0) and 28 days after vaccination.

	Group 2 TCV + MCV-A (*n* = 50)	Group 3 MCV-A + IPV (*n* = 51)
Day 0 SBA titre ≥128	24^a^ (49.0%, 34.4–63.7%)	10 (19.6%, 9.8–33.1%)
Day 28 SBA titre ≥128	48^b^ (100.0%, 92.6–100.0%)	49^a^ (98.0%, 89.4–100.0%)
Day 0 geometric mean titre	34.3^a^ (14.7–80.4)	6.4 (3.3–12.7)
Day 28 geometric mean titre	13 385.0^b^ (9784.4–18 310.6)	9410.1^a^ (6009.2–14 735.9)

Data are *n* (%, 95% confidence interval) or mean (95% confidence interval). *n* = number of participants. IPV, inactivated polio vaccine; MCV-A, group A meningococcal conjugate vaccine; SBA, serum bactericidal antibody; TCV, typhoid conjugate vaccine.

a One participant missing titre.

b Two participants missing titre.

Anti-tetanus IgG antibody titres for groups 1, 2, and 3 were almost all above the threshold for short-term immunity and about half of the participants were above the threshold for long-term immunity (Supplementary Material Table A3 and Figure 2C). Post-vaccination, only one participant, in group 1 (TCV + IPV group), was below the threshold for short-term immunity, while all other participants exceeded thresholds for long-term immunity. Analyses by sex showed similar results (data not shown).

## Discussion

Among 15–23-month-old children in Burkina Faso, a single-dose of TCV was safe and well-tolerated when co-administered with routine MCV-A and MR vaccines. TCV did not interfere with the immune response to MCV-A and demonstrated tolerability and immunogenicity in this population of children. These results provide evidence for public health officials and policymakers who are considering the implementation of routine TCV administration in endemic areas, particularly where MCV-A is routinely given.

While this study was small, the safety and reactogenicity profiles are reassuring and consistent with those reported in studies performed in other parts of the world, including India and Pakistan, where over 200,000 doses were administered to children 6 months to 15 years of age during vaccination campaigns in 2018 (World Health Organization, 2019a). Favourable safety has also been documented in studies of TCV in children 9 months to 16 years of age in Nepal (Shakya et al., 2019), children 2 years of age and older in India (Mohan et al., 2015), and in adults in the United Kingdom (Jin et al., 2017). The administration of TCV that fuses the Vi polysaccharide to a tetanus toxoid carrier increases the cumulative exposure of children to tetanus toxoid after repeated vaccinations during infancy and increases simultaneous exposure with co-administration of MCV-A, a separate tetanus toxoid-containing vaccine. In theory, this increased exposure to tetanus toxoid might increase the risk of reactogenicity to TCV, but this was not observed in this study. In contrast, the addition of a tetanus-containing vaccine may serve to increase long-term immunity to tetanus, providing an added benefit of TCV.

Importantly, TCV did not interfere with MCV-A immunogenicity, as no differences were seen in participants who received MCV-A with and without TCV co-administration. As both vaccines are conjugated to a tetanus toxoid carrier, simultaneous administration could theoretically lead to a dominant serological response to either vaccine. However, no interference with immune responses to either vaccine was documented, confirming the robust immunogenicity profile of TCV and MCV-A and preservation of each vaccine’s capacity to combat communicable disease threats in the region.

TCV generated a robust antibody response 28 days after vaccination. These results are consistent with those of other studies that have evaluated TCV immunogenicity (Shakya et al., 2019, Mohan et al., 2015, Jin et al., 2017, Lin et al., 2001). Using the same TCV, a recent study in Nepalese children 9 months to 16 years of age reported a GMT of 2038 EU/mL at 28 days after a single-dose vaccination (Shakya et al., 2019). This study used the same Vi antibody assay kit employed to measure responses, and both populations showed a robust seroconversion rate. While there is no correlate of protection for TCV, the results from this study showed similar or higher antibody responses compared to those in Nepalese children, in whom efficacy has been demonstrated, which is reassuring. In addition, a single-dose TCV reduced typhoid incidence in Zimbabwe after a mass vaccination outbreak response campaign was conducted in 2019 among children aged 6 months to 15 years (Olaru et al., 2019).

In 2018, the WHO recommended TCV for children in countries where the typhoid fever burden is high and encouraged countries to consider TCV integration into routine childhood immunization schedules (World Health Organization, 2019b). Until now, there were no data on TCV co-administration with routine MCV-A to guide public health officials and policymakers on decisions for vaccine introduction. This represents the first study of TCV in West Africa, an area with a significant risk of typhoid fever, especially in young children. The concurrent risk of meningococcal A disease in this population warrants protection from both illnesses, and the study results confirm that the co-administration of TCV does not diminish the response to MCV-A in Burkinabe children. TCV can therefore be routinely administered with MCV-A to young children.

This study is also novel in evaluating TCV immunization with routine vaccination at the 15-month visit. Options for routine TCV immunization include visits at 9 or 15 months of age. Other TyVAC studies of TCV administration at the routine 9-month vaccination visit are underway and will add to existing data for consideration by countries as they decide to add TCV to routine vaccination schedules. With the increasing number of vaccines given at 9 months, countries now have the option of either 9 or 15 months for TCV delivery.

## Data sharing

After publication, the authors will provide individual participant data that underlie the results reported in this article, after de-identification (text, tables, figures, and appendices), to researchers who provide a methodologically sound proposal with approved aims. The study protocol will also be made available. Proposals should be directed to mlaurens@som.umaryland.edu; to gain access, data requestors will need to sign a data access agreement. Proposals may be submitted up to 36 months following article publication. After 36 months, the data will be available in the university’s data warehouse, but without investigator support other than deposited metadata.

## Funding source

This trial was funded in part by a grant from the Bill & Melinda Gates Foundation.

## Ethical approval

The study was approved by the ethics committees in Burkina Faso (Comité d’Ethique pour la Recherche en Santé (CERS), Ouagadougou, Burkina Faso) and Maryland, USA (Institutional Review Board, University of Maryland, Baltimore), and by the Regulatory Authority in Burkina Faso (L’Agence Nationale de Régulation Pharmaceutique, Ouagadougou, Burkina Faso).

## Conflict of interest

The authors have no conflict of interest.

## Author contributions

KMN, SBS, MBL, and ETR conceived the study, developed the protocol and SOPs, and managed the ethical submissions. AO, NB, MS, AT, AK, and GDB recruited participants and performed participant follow-up procedures. INO, AD, and MO collected and processed clinical specimens for immunogenicity and generated anti-Vi and anti-tetanus titres. IS and AH managed the study vaccines, performed the randomization, and supervised the injections. JKT designed and implemented the REDCap data capture and management procedures to support the trial. YL developed the statistical analysis plan and conducted the analyses. SD conducted the analyses and created the figures and tables. LPJ created the figures and tables and edited the manuscript. All authors read and approved the final manuscript.
