# Supplementary material for: Safety and immunogenicity of co-administration of meningococcal type A and measles–rubella vaccines with typhoid conjugate vaccine in children aged 15–23 months in Burkina Faso
Source: Int J Infect Dis. 2021 Jan;102:517–23. doi: 10.1016/j.ijid.2020.10.103 (PMC7762715; doi:10.1016/j.ijid.2020.10.103)
Supplement: Supplementary file 1 [file mmc1.docx]

Supplementary tables

|  |  | Male | | Female | | p-value* |
| --- | --- | --- | --- | --- | --- | --- |
|  |  | **n** | **GMT (95% CI)** | **n** | **GMT (95% CI)** |  |
| Group 1:TCV + IPV (delayed MCV-A dose) | Day 0 | 29 | 4.4 (3.6-5.3) | 19 | 6.1 (3.8- 9.8) | 0.28 |
|  | Day 28 | 28 | 2307.4 (1058.3-5030.7) | 19 | 3574.8 (1613.4-7920.9) | 0.73 |
| Group 2: TCV + MCV-A | Day0 | 27 | 5.0 (3.5-7.1) | 23 | 4.3 (3.5-5.4) | 0.52 |
|  | Day 28 | 27 | 4051.4 (3022.9-5429.9) | 23 | 3340.5 (1764.2-6325.2) | 0.66 |
| Group 3: MCV-A + IPV | Day 0 | 20 | 4.5 (3.1- 6.7) | 31 | 5.0 (3.8- 6.8) | 0.62 |
|  | Day 28 | 20 | 5.6 (3.7- 8.6) | 31 | 5.1 (3.7- 7.0) | 0.90 |

n=number of participants. GMT=geometric mean titre. CI=confidence interval.

*Comparison of GMT between males and females using two sample t-test on log_10_ transformed data.

**Table A1: Anti-Vi IgG antibody geometric mean titres before vaccination (day 0) and 28 days after vaccination, by sex**

|  |  | Male | | Female | |  |
| --- | --- | --- | --- | --- | --- | --- |
|  |  | **n** | **GMT (95% CI)** | **n** | **GMT (95% CI)** | **p- value*** |
| Group 2: TCV + MCV-A | Day 0 | 27 | 49.5 (16.3-150.6) | 22 | 21.9 (6.4, 75.5) | 0.34 |
|  | Day 28 | 26 | 15953.0 (10192.3-24969.6) | 22 | 10878.0 (7334.6, 16132.5) | 0.36 |
| Group 3: MCV-A + IPV | Day 0 | 20 | 6.3 (2.2-17.7) | 31 | 6.5 (2.7, 15.7) | 0.96 |
|  | Day 28 | 19 | 8496.4 (4414.3- 16353.3) | 31 | 10018.0 (5577.5, 17994.2) | 0.67 |

n=number of participants. GMT=geometric mean titre. CI=confidence interval.

*Comparison of GMT between males and females using two sample t- test on log_10_ transformed data.

**Table A2: Serum bactericidal antibody geometric mean titres before vaccination (day 0) and 28 days after vaccination, by sex**

|  | **Group 1:**  **TCV + IPV (delayed MCV-A)** | | **Group 2:**  **TCV + MCV-A** | | **Group 3:**  **MCV-A + IPV** | |
| --- | --- | --- | --- | --- | --- | --- |
|  | **n/N** | **% (95% CI)** | **n/N** | **% (95% CI)** | **n/N** | **% (95% CI)** |
| Short-term immunity (>0.1 IU/mL) |  |  |  |  |  |  |
| Day 0 | 46/48 | 95.8 (85.8-99.5) | 50/50 | 100.0 (92.9-100.0) | 51/51 | 100.0 (93.0-100.0) |
| Day 28 | 46/47 | 97.9 (88.7-100.0) | 50/50 | 100.0 (92.9-100.0) | 51/51 | 100.0 (93.0-100.0) |
| Long-term immunity (>1.0 IU/mL) |  |  |  |  |  |  |
| Day 0 | 24/48 | 50.0 (35.2-64.8) | 21/50 | 42.0 (28.2-56.8) | 29/51 | 56.9 (42.3-70.7) |
| Day 28 | 46/47 | 97.9 (88.7-100.0) | 50/50 | 100.0 (92.9-100.0) | 51/51 | 100.0 (93.0-100.0) |

n=number of participants. N=total number. CI=confidence interval. IU=international unit.

**Table A3: Anti-tetanus IgG antibody immunogenicity before vaccination (day 0) and 28 days after vaccination**
